# Declined adipogenic potential of senescent MSCs due to shift in insulin signaling and altered exosome cargo

**DOI:** 10.3389/fcell.2022.1050489

**Published:** 2022-11-17

**Authors:** Elizaveta Voynova, Konstantin Kulebyakin, Olga Grigorieva, Ekaterina Novoseletskaya, Natalia Basalova, Natalia Alexandrushkina, Mikhail Arbatskiy, Maxim Vigovskiy, Anna Sorokina, Anna Zinoveva, Elizaveta Bakhchinyan, Natalia Kalinina, Zhanna Akopyan, Vsevolod Tkachuk, Pyotr Tyurin-Kuzmin, Anastasia Efimenko

**Affiliations:** ^1^ Faculty of Medicine, Lomonosov Moscow State University, Moscow, Russia; ^2^ Institute for Regenerative Medicine, Medical Research and Education Center, Lomonosov Moscow State University, Moscow, Russia

**Keywords:** senescence, extracellular vesicles (EVs), insulin signaling, adipogenic potential, MSCs (mesenchymal stromal cells)

## Abstract

Multipotent mesenchymal stromal cells (MSCs) maintain cellular homeostasis and regulate tissue renewal and repair both by differentiating into mesodermal lineage, e.g., adipocytes, or managing the functions of differentiated cells. Insulin is a key physiological inducer of MSC differentiation into adipocytes, and disturbances in MSC insulin sensitivity could negatively affect adipose tissue renewal. During aging, regulation and renewal of adipose tissue cells may be disrupted due to the altered insulin signaling and differentiation potential of senescent MSCs, promoting the development of serious metabolic diseases, including metabolic syndrome and obesity. However, the potential mechanisms mediating the dysfunction of adipose-derived senescent MSC remains unclear. We explored whether aging could affect the adipogenic potential of human adipose tissue-derived MSCs regulated by insulin. Age-associated senescent MSCs (isolated from donors older than 65 years) and MSCs in replicative senescence (long-term culture) were treated by insulin to induce adipogenic differentiation, and the efficiency of the process was compared to MSCs from young donors. Insulin-dependent signaling pathways were explored in these cells. We also analyzed the involvement of extracellular vesicles secreted by MSCs (MSC-EVs) into the regulation of adipogenic differentiation and insulin signaling of control and senescent cells. Also the microRNA profiles of MSC-EVs from aged and young donors were compared using targeted PCR arrays. Both replicatively and chronologically senescent MSCs showed a noticeably decreased adipogenic potential. This was associated with insulin resistance of MSCs from aged donors caused by the increase in the basal level of activation of crucial insulin-dependent intracellular effectors ERK1/2 and Akt. To assess the impact of the paracrine cross-talk of MSCs, we analyzed microRNAs profile differences in MSC-EVs and revealed that senescent MSCs produced EVs with increased content of miRNAs targeting components of insulin-dependent signaling cascade PTEN, MAPK1, GAREM1 and some other targets. We also confirmed these data by differentiation of control MSCs in the presence of EVs from senescent cells and *vice versa*. Thus, aging attenuated the adipogenic potential of MSCs due to autocrine or paracrine-dependent induction of insulin resistance associated with the specific changes in MSC-EV cargo.

## Introduction

Aging and obesity are major risk factors for the most common chronic diseases, including diabetes, cardiovascular disease, hepatic steatosis, and cancer, which promotes the importance of studies related to the age-associated adipose tissue dysfunctions. Adipose tissue is currently considered as the largest energy storage depot and a powerful endocrine organ (Kershaw and Flier 2004; Galic et al., 2010). Adipose tissue inflammation and dysfunction are associated with age- and obesity-related insulin resistance, metabolic syndrome and diabetes, arterial hypertension and atherosclerosis, but the exact mechanisms underlying these relationships are still elaborating (Guilherme et al., 2008; Hajer et al., 2008; Palmer et al., 2019). To function properly over lifetime, the adipose tissue should be constantly renewed, and its turnover reaches about 10% of cells per year (Spalding et al., 2008; Arner et al., 2010). Retardation of adipose tissue renewal leads to the accumulation of hypertrophic adipocytes which maintain the ability for lipid storage but attenuate the endocrine functioning (Hammarstedt et al., 2018).

In adults the renewal of adipose tissue throughout the life is provided by differentiation of multipotent mesenchymal stromal cells (MSCs). Insulin is a key physiological inducer of MSC differentiation into adipocytes (Zhang et al., 2012). Disturbances in MSC insulin sensitivity could negatively affect adipose tissue renewal and promote the development of adipocyte hypertrophy and metabolic disorders (Kim et al., 2014). There is a vast amount of evidence that aging and obesity are associated with an increase in the senescent cell burden in multiple organs, including those in MSC populations, which could seriously affect adipose tissue renewal (Childs et al., 2015; Cárdenes et al., 2018; Neri and Borzì 2020; Narasimhan et al., 2021).

Senescence, a cellular response to endogenous and exogenous stresses limiting the proliferation of damaged and dysfunctional cells, markedly contributes to both physiological aging and age-related diseases (van Deursen 2014; Childs et al., 2015; McHugh and Gil 2018). Cell senescence can be induced by harmful stimuli such as DNA damage, telomere shortening, oncogene activation, metabolic stress, epigenetic changes, and mitochondrial dysfunction (Liu et al., 2020). One of the first established models of cellular senescence was replicative senescence described in long-term cultured cells and associated with the shortening of telomeres, DNA structures located at the ends of eukaryotic chromosomes and protecting them from degradation, as well as activation of cell cycle inhibitors such as p16/Ink4a, p21/Cip1 and p53. The senescent cells are also routinely characterized by enlarged cell size, flattened morphology, and enhanced senescence-associated (SA)-β-galactosidase activity. However, the most important hallmark of MSC senescence are the functional alterations caused by metabolic, genetic, epigenetic, transcriptional, and translational changes (see the detailed review by Neri and Borzì 2020). In addition, these cells acquire a senescence-associated secretory phenotype (SASP) involving the secretion of factors that can affect the behavior of neighboring cells *via* autocrine/paracrine mechanisms and reprogram the microenvironment toward the prosenescent state (Borodkina et al., 2018; Campisi et al., 2019). These SASP factors could directly mediate adipose tissue dysfunction and insulin resistance in peripheral tissues promoting metabolic disorders, including the onset of type II diabetes mellitus (Schafer et al., 2017; Narasimhan et al., 2021). The accumulating data indicates that during aging of an organism, senescent MSCs imply an impairment of stem cell functions contributing to the progressive decrease in tissue renewal and regeneration (Efimenko et al., 2014; Schimke et al., 2015; Neri and Borzì 2020; Sagaradze et al., 2020). Thus, it was shown that adipogenic differentiation was impaired in replicative senescent human MSCs from subcutaneous adipose tissue (Mitterberger et al., 2014).

Therefore, during aging, regulation and renewal of adipose tissue cells may be disrupted due to the altered insulin signaling and differentiation potential of senescent MSCs, promoting the development of serious metabolic diseases, including metabolic syndrome and obesity. In this study we explored whether senescence could affect the adipogenic potential of human adipose tissue-derived MSCs regulated by insulin and what potential mechanisms could underlie this dysfunction.

## Material and methods

### Cell culture

Primary cell lines of MSC isolated from abdominal adipose tissue of healthy young (*n* = 6, median of age 38 years) and aged (*n* = 10, age >65 years, median of age 69 years) donors were obtained from the biobank of the Institute for Regenerative Medicine, Medical Research and Education Center, Lomonosov MSU, collection ID: MSU_MSC_AD. All procedures performed with tissue samples from patients were in accordance with the Declaration of Helsinki and approved by the Ethic Committee of Medical Research and Education Center, Lomonosov Moscow State University (IRB00010587), protocol #4 (date of approval 4 June 2018) and protocol #9 (date of approval 29 October 2018), and all donors provided informed consent. Cells were cultured in Mesenchymal Stem Cell Basal Medium containing AdvanceSTEM medium (HyClone, Logan, UT, USA) supplemented with 10% Mesenchymal Stem Cell Growth Supplement (HyClone, Logan, UT, USA), 1% Penicillin/Streptomycin solution (Gibco, Logan, UT, USA), 1% L-glutamine (Gibco, Logan, UT, USA) under an atmosphere of 5% CO2 at 37°C. Cells were passaged at 70–80% confluency using Versen solution (Paneco, Russia) and HyQTase solution (HyClone, Logan, UT, USA), with a subcultivation ratio of 1:3. For the experiments, MSCs cultured up to the 4th-5th passages were used in case of control MSCs and MSCs from aged donors while up to the 9th-12th passages in case of induced replicative senescence.

### Extracellular vesicles isolation

MSCs isolated from the old donors (age-associated senescent MSCs), and MSC isolated from the young healthy donors used either at 2-5 passages (control MSCs) or at 8–12 passages (replicative senescent MSCs) were cultured to approximately 90%–100% confluence before being washed three times with Hanks’ buffer solution (Paneco, Russian Federation) for 10 min each. Then, the cells were incubated for 48 h with DMEM low glucose without Phenol Red (Gibco, USA). Conditioned medium was harvested and centrifuged for 10 min at 300 g to remove cell debris. EVs were isolated from MSC conditioned medium using Amicon filter (300 kDa, Sartorius, Germany). All samples were stored at −80°C. The particle size and concentration of EV samples were analyzed *via* nanoparticle tracking analysis (NTA; ZetaView, Particle Metrix), the morphology was visualized *via* transmission electron microscopy (TEM), and exosomal markers in EVs or MSC lysates were evaluated *via* immunoblotting as described in (Basalova et al., 2020).

### Cell senescence characterization

Variable biomarkers of cellular senescence were explored in isolated MSCs. Proliferation of MSCs was analyzed using IncuCyte^®^ ZOOM Live Cell Analysis System (Essen Bioscience, USA) and time-lapse acquisition was carried out every hour for 72 h. The device’s built-in software allows to estimate the area occupied by cells by applying a “mask” to the obtained images and thus calculating the percentage of cell culture confluency. The increase in confluence directly correlates with the increase in the number of cells, which allows us to judge the growth rate of cell culture by calculated parameters including population doubling time (PDT = t/[log2(N/N0)], where t—time of cell growth, N—area occupied by cells at the beginning of time interval, N0—area occupied by cells at the end of time interval) and lag phase. To evaluate the telomere length MSCs were thoroughly washed and lysed in RLT buffer (Qiagen), then processed using Absolute Human Telomere Length Quantification qPCR Assay Kit (ScienCell, USA) according to the manufacturer’s instructions. To measure the number of p21-positive cells MSCs were fixed with 4% paraformaldehyde solution (Panreac, Barselona, Spain) at room temperature for 10 min and incubated with 0.2% triton ×100 (Sigma) solution at RT for 10 min. Further, MSCs were incubated for 1 h in 1% bovine serum albumin (BSA, Sigma) and 10% normal goat serum (Abcam, Cambridge, UK) solution at room temperature to block the nonspecific interaction of antibodies. Subsequently, the samples were incubated with primary polyclonal rabbit antibody for p21Waf1/Cip1 (Cell Signaling, 2947S) or rabbit polyclonal IgG (Biolegend, San Diego, CA, USA, 910801) in 1% BSA solution at +4° overnight. Then, samples were incubated with fluorescence-labeled goat anti-rabbit secondary antibodies (A11034, Invitrogen) at room temperature for 1 h. Cell nuclei were labeled with DAPI (DAKO). Samples were analyzed with a Leica DM6000B fluo-rescent microscope equipped with a Leica DFC 360FX camera (Leica Microsystems GmbH, Wetzlar, Germany). The percentage of p21-positive MSCs was evaluated. Evaluation of β-galactosidase activity was performed with the Senescence β-Galactosidase Staining Kit (Cell Signaling, Danvers, MA, USA) according to the manufacturer’s recommendations. SASP components interleukine 6 (IL-6), monocyte chemoattracting protein 1 (MCP-1) and plasminogen activator inhibitor 1 (PAI-1) were measured in MSC conditioned media collected in 72 h of conditioning by ELISA (R&D Systems) according to the manufacturer’s recommendations.

### Adipogenic differentiation

MSCs isolated from the aged donors (age-associated senescent MSCs), and MSCs isolated from the young healthy donors used either at 2-5 passages (control MSCs) or at 8–12 passages (replicative senescent MSCs) were cultured in Mesenchymal Stem Cell Basal Medium supplemented with 10% Mesenchymal Stem Cell Growth Supplement into 12-well plates until confluency. Then cells were directed into adipogenic differentiation using adipogenic differentiation cocktail containing DMEM low glucose (HyClone, USA) supplemented with 10% of FBS (HyClone), 1% Penicillin/Streptomycin solution (Gibco, Logan, UT, USA), 1 µМ dexamethasone, 200 µM insulin and 0.5 mM 3-isobutyl-1-methylxantine (IBMX, Millipore, USA) for 14 days. Medium was refreshed every 2–3 days. To evaluate the effect of EVs on adipogenic differentiation we pretreated cells with EVs (1–3*10^9 particle per ml) secreted by control or senescent MSCs dissolved in DMEM low glucose a day before induction of adipogenic differentiation. Next day we induced adipogenic differentiation by adding the components of the differentiation cocktail supplemented by EVs to the cells and then refreshing the differentiation medium with EVs every 2–3 days. Accumulation of intracellular lipid droplets over time of differentiation was visualized using transmitted light microscopy. Inverted widefield microscope Nikon Eclipse Ti-E equipped with an objective CFI Plan Fluor DLL 10X/0.3 (Nikon, Tokyo, Japan) and with digital cooled monochrome CCD camera Nikon DS-Qi1 (Nikon, Tokyo, Japan) was used. We used the simultaneous measuring of 12 × 12 fields of view in Large Image mode to increase the number of analyzed cells. Images were analyzed using NIS-Elements (Nikon) and ImageJ software (NIH, Bethesda, MD, USA). After the end of differentiation neutral lipids were additionally stained with Nile Red (Sigma, Merck Millipore, USA) and visualized into the same fields of view. mRNA levels of adipose differentiation master-genes were evaluated 14 days after the induction of differentiation.

### Western blotting

Cell protein samples were obtained *via* cell lysis in a sample buffer (62.5 mM Tris-HCl pH 6.8, 2.5% SDS, 0.002% Bromophenol Blue, 5% β-mercaptoethanol, 10% glycerol). Proteins were divided by the SDS-PAGE method. Afterward, proteins were transferred from polyacrylamide gel to the PVDF membrane by Western blotting. TBS containing 0.1% Tween-20 and 5% BSA (PanEco, Moscow, Russia) was used to prevent non-specific binding. The next step was overnight staining of the membrane with protein-specific antibodies to total Akt [Akt (pan) (C67E7) Rabbit mAb #4691; Cell Signaling Technology Inc., Danvers, MA, United States], phosphorylated T308 Akt [Anti p-Akt (Thr308) (244F9) Rabbit mAb #4056; Cell Signaling Technology Inc., Danvers, MA, United States], total ERK [p44/42 MAPK (Erk1/2) (137F5) Rabbit mAb #4695; Cell Signaling Technology Inc., Danvers, MA, United States], phospho-ERK [Phospho-p44/42 MAPK (Erk1/2) (Thr202/Tyr204) (E10) Mouse mAb #9106; Cell Signaling Technology Inc., Danvers, MA, United States] and Vinculin [Anti Vinculin Rabbit antibody V4139; Sigma-Aldrich]. Unbound antibodies were then washed away, and the rest were incubated with antibodies for total rabbit immunoglobulins conjugated with peroxidase [P-GAR Iss; IMTEK, Moscow, Russia] or with antibodies for total rabbit immunoglobulins conjugated with peroxidase [Goat Anti-Mouse IgG Antibody, HRP conjugate, Sigma-Aldrich] for 1 h. Amplified chemiluminescence was used as a visualization method with a Clarity ECL detection kit (Bio-Rad). Image registration was carried out using the ChemiDoc Touch gel documenting system (Bio-Rad). Image analysis and volume measurements were performed using the Image Lab Software (Bio-Rad). Total Akt and ERK staining volume readings were normalized to the respective vinculin level, and then volume readings for p-Akt or p-ERK were compared with respective normalized Akt or ERK.

### RT-PCR

The RNeasy Mini Kit (Qiagen) was used to extract RNA. cDNA was synthesized from 500 ng RNA with the MMLV Reverse Transcription Kit (Evrogen, Moscow, Russia) according to the manufacturer’s instructions. The relative expression of gene-markers adipogenic differentiation PPARγ and adiponectin was analyzed by quantitative real-time PCR. The following equipment were used: qPCR mix-HS SYBR + LowROX (Evrogen) reagents and CFX96 Touch Real-Time PCR Detection System (Bio-Rad, Hercules, CA, United States). The gene of 60S Ribosomal protein P0 (*RPLP0*) was used as a housekeeping gene. Quantification and normalization of expression levels of the target genes and the reference gene (*RPLP0*) were calculated using the comparative threshold cycle (CT) method. Primers for PCR were picked using the NCBI Primer Designing Tool. Primer sequences are presented below:

60S Ribosomal protein P0 (RPLP0), For: GCT​GCT​GCC​CGT​GCT​GGT​G, Rev: TGG​TGC​CCC​TGG​AGA​TTT​TAG​TGG, 130bp. Adiponectin (ADIPOQ), For: GAC​CAG​GAA​ACC​ACG​ACT​CA, Rev: TTT​CAC​CGA​TGT​CTC​CCT​TAG​G, 199bp. Peroxisome proliferator-activated receptor gamma (PPARγ), For: TCA​GGT​TTG​GGC​GGA​TGC, Rev: TCA​GCG​GGA​AGG​ACT​TTA​TGT​ATG, 147bp.

### EV microRNA PCR analysis and bioinformatics

MicroRNAs from EVs secreted by control or senescent MSCs were isolated using miRNeasy Mini Kit (Qiagen) according to the manufacturer’s instructions. RNA was quantified and qualified using Nanodrop spectrophotometer (Thermo Scientific) by 260/230 nm ratio. Reverse transcription was performed using miScript II RT Kit (Qiagen) in accordance with the manufacturer’s protocol. Real-time PCR was performed using specific miScript miRNA PCR Array (Qiagen) and miScript SYBR Green PCR Kit (Qiagen) containing 2x QuantiTect SYBR Green PCR Master Mix and universal microRNA primer on QuantStudio 5 Real-Time PCR System (Thermo Fisher Scientific). The expression levels of microRNAs were calculated relative to those of house-keeping microRNAs SNORD61 (two variants), SNORD95, SNORD96a, RNU6 (included in PCR array) using the comparative ΔCT method.

The analysis of the miRNA array was performed using the sRNAtoolbox web-server (parameters: miRNAs: miRBase v22; ncRNA: (Ensembl release 91 (ncRNA); length of the seed: 20; number of allowed mismatches: 2; phred score: 20). Using the TargetScan7.2, HMDD, miR2Disease, miRwayDB databases, miR’s associated with the senescence process and their representation in MSC-EV were analyzed. Prediction of miRNA functions was performed using the Gene Ontology (GO) and Kyoto Encyclopedia of Genes and Genomes (KEGG) databases. GO and KEGG gene clustering was performed using the David 6.8 database. Quality control, mapping and normalization of the miRNA array was performed using the BrowserGenome 1.0 web-based deep-sequencing data-analysis platform (hg38 GENCODE 22 genome). Keyword analysis was performed using our own bash scripts with following commands: while read line; grep; awk; sed; sort (uniq). Common predicted microRNA targets were analyzed using miRNet, miRBase and miRDB databases.

After obtaining the lists of targets for identified microRNAs, using the CytoScape platform, we analyzed these lists and created interaction maps, where microRNAs were located as nodes and microRNA targets as edges. We also showed on these maps the distribution of such an important indicator as the target score. All possible targets have a target score between 50 and 100. The closer it is to 100, the more reliable this prediction can be. The result of the search for targets is most often ordered precisely by the value of the target score. As recommended in the literature, a target for which a target score >80 is likely to be real. If the score is below 60, then additional evidence should be sought confirming the participation of this target.

### Statistical analysis

Statistical analysis was performed using SigmaPlot 12.5 software (Systat Software Inc., San Jose, CA, USA). Data were assessed for normality of distribution using the Shapiro-Wilk test. Values are expressed as mean ± standard error of the mean (SEM). Comparison of two independent groups was performed by Student t-test for normally distributed data and Mann–Whitney U-criteria (M-U test) for not normally distributed data. Multiple comparisons were made using the Kruskal-Wallis test (one-way ANOVA on ranks) with subsequent application of Dunn criteria. Statistical significance was defined as *p*-value <0.05.

## Results

### Senescent MSCs demonstrate impaired adipogenic potential

**FIGURE 1:**
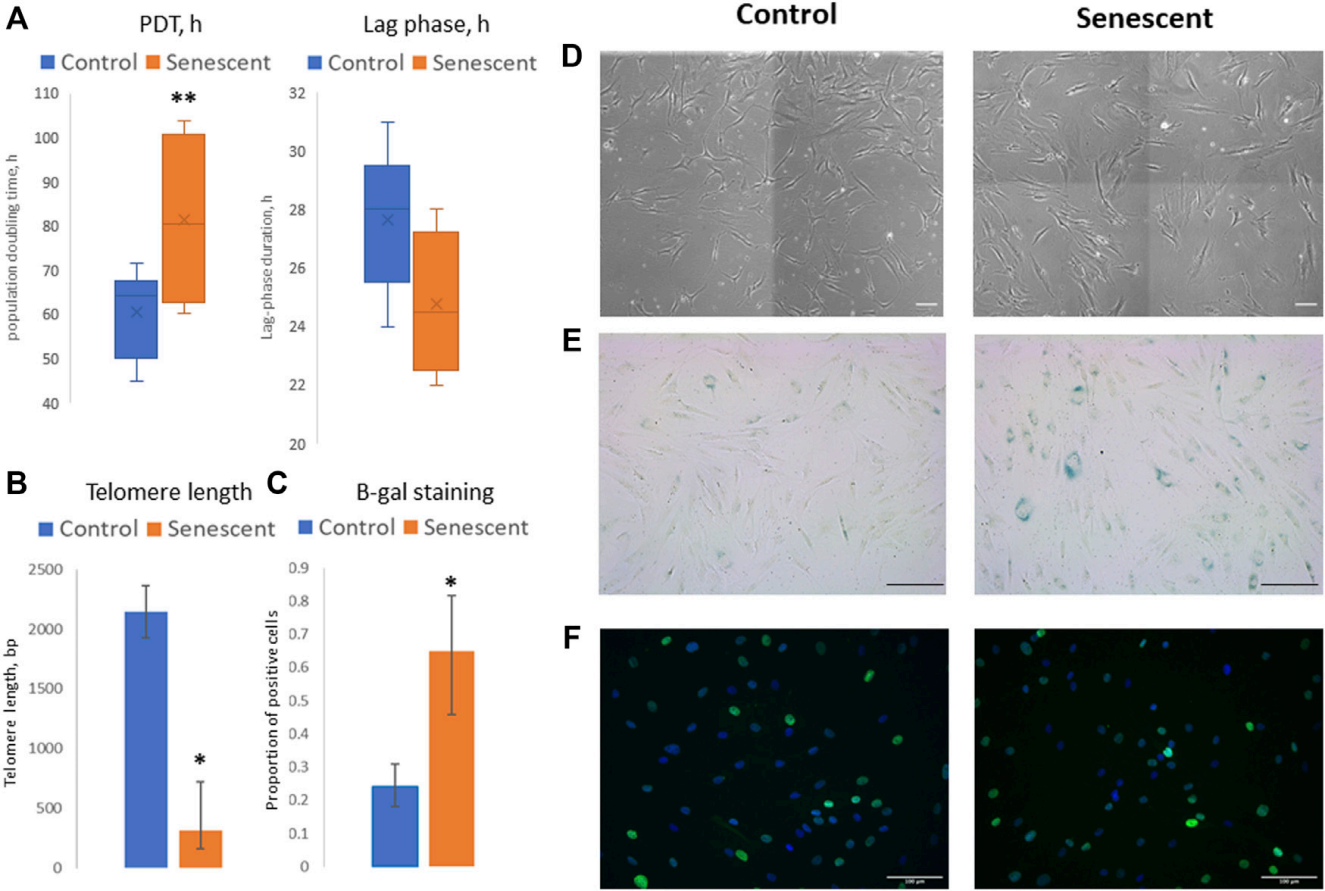
Senescent biomarkers in MSCs isolated from young and aged donors. **(А)**—population doubling time (PDT, hours) and lag phase duration (hours) measured in Incucyte ZOOM system; **(B)**—telomere length (bp); **(C)**—proportion of cells with positive staining for beta-galactosidase (b-gal); **(D–F)–**representative microphotographs of MSCs isolated from young and aged donors: **(D)**—cell morphology in culture, phase-contrast images; **(E)**—b-gal staining, scale bar = 100 μm; **(F)**—immunocytochemical staining for p21 expression (green), nuclei are stained with DAPI). Control–MSCs isolated from young donors, senescent–MSCs isolated from aged donors. Mean ± SE, *p < 0.05, **p = 0.057.

To evaluate the molecular mechanisms of the changes in adipogenic potential of MSC during the development of cell senescence, we isolated MSCs from the adipose tissue of aged patients considering this model as physiological aging. Control cells were isolated from the adipose tissue samples obtained from young healthy donors. Additionally, MSCs from the young donors cultured up to 9–12 passages were used as a model of replicative senescence. First, the main biomarkers of cellular senescence were analyzed in MSCs, namely: proliferation potential, telomere length, expression of cell cycle inhibitors (p21), beta-galactosidase staining, and secretion of some components of SASP (Figure 1). Average PDT of cells derived from the aged donors was higher compared to MSCs from the young donors (81.2 h vs. 60.5 h), while the duration of a lag phase was slightly shorter (24.8 h vs. 27.7 h) (Figure 1A). It should be noted that we analyzed the heterogeneous primary cell population, reflected by variable MSC morphology as both large, flattened cells with morphological signs of senescence and smaller spindle-like cells were observed in culture; however, the latter type prevailed mostly in MSCs from the young donors (Figure 1D). Thus, a shorter lag phase (earlier appearance of mitotic cells in culture) could indicate the presence of a small proliferating subpopulation contributed to the cell population growth. However, the total proliferation potential of MSCs from the aged donors was lower which was confirmed by the shorter telomeres in these cells (Figure 1B) and a slight increase in p21 expression (Figure 1F). Expression of beta-galactosidase was also higher in MSCs from the aged donors compared to control cells (Figures 1C,E). These cells acquired SASP resulting in increased secretion of IL-6 (38.6 vs. 11.7 ng/ml), MCP-1 (304.9 vs. 6.1 ng/ml), and PAI-1 (4.2 vs. 1.1 ng/ml) compared to MSCs from the young donors.

**FIGURE 2:**
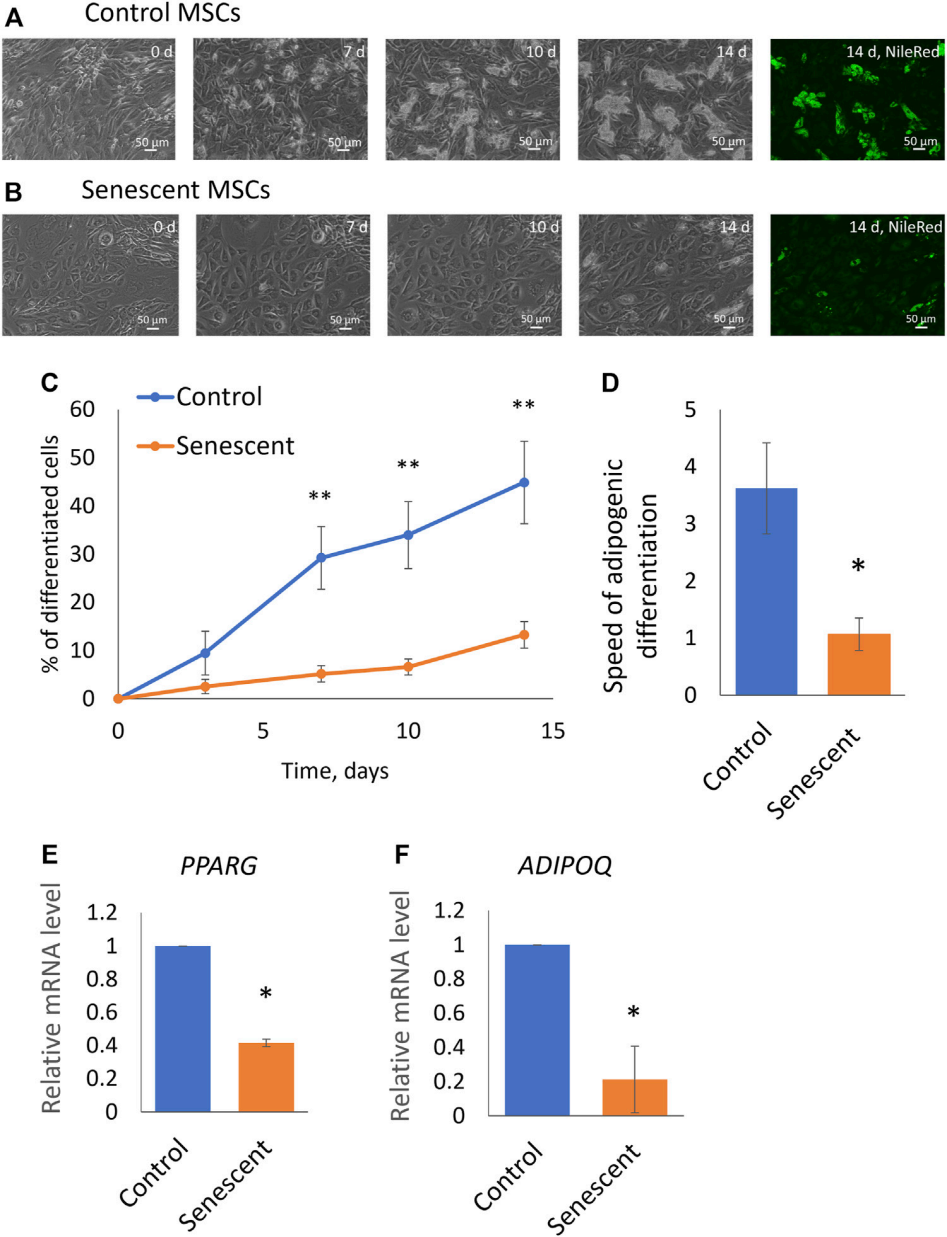
Senescent MSCs demonstrated reduced adipogenic potential. **(A,B)**, Representative phase-contrast images of the time dynamics of differentiation of control **(A)** and senescent **(B)** cells on 0, 7, 10, and 14 days in the same fields of view, and staining of lipid droplets in these cells with Nile Red dye in the end point (14th day). **(C)**. Dynamics of increase in the number of cells accumulating fat drops during adipogenic differentiation of MSCs, *n* = 6–7; **(D)**. The tangent of the slope of the increase in the number of differentiating cells (rate of differentiation, increase in percent of differentiated cells per day), *n* = 7–9; **(E,F)**. Expression level of markers of adipogenic differentiation PPARγ (*PPARG*) **(E)** and adiponectin (*ADIPOQ*) **(F)** on 14 days of adipogenic differentiation, *n* = 3–7. Mean ± SE. *p < 0.05, **p < 0.01.

To find out how aging affects the adipogenic potential of MSCs, we stimulated the differentiation of control and senescent MSCs into adipocytes. The standard adipogenic differentiation cocktail includes hormonal differentiation inducers (insulin and dexamethasone) and also indomethacin, a direct activator of the transcriptional factor PPARg, which is the master regulator of adipogenic differentiation. Our study aimed to show the involvement of hormonal signalling in changes of the adipogenic potential of MSCs during aging, that is why in this work, we did not use indomethacin. We have induced adipogenic differentiation of control and senescent cells with a reduced cocktail containing insulin, dexamethasone, and IBMX. As seen in Figure 2, aging reduced the efficiency of MSC differentiation in the adipocyte direction. Both the efficiency (Figures 2A–C) and the rate (Figure 2D) of adipogenic differentiation was decreased. The induction of replicative senescence leads to a similar decrease in both the efficiency and the rate of adipogenic differentiation (Supplementary Figures S1, S2). In addition, we measured the expression of marker genes of adipogenic differentiation. As shown in Figures 2E,F, expression of PPARg as well as key adipokine adiponectin was reduced in senescent cells (Figure 2E). Thus, aging reduces the adipogenic potential of MSCs in adipose tissue.

### Intracellular signaling in senescent MSCs indicate the development of insulin resistance

**FIGURE 3:**
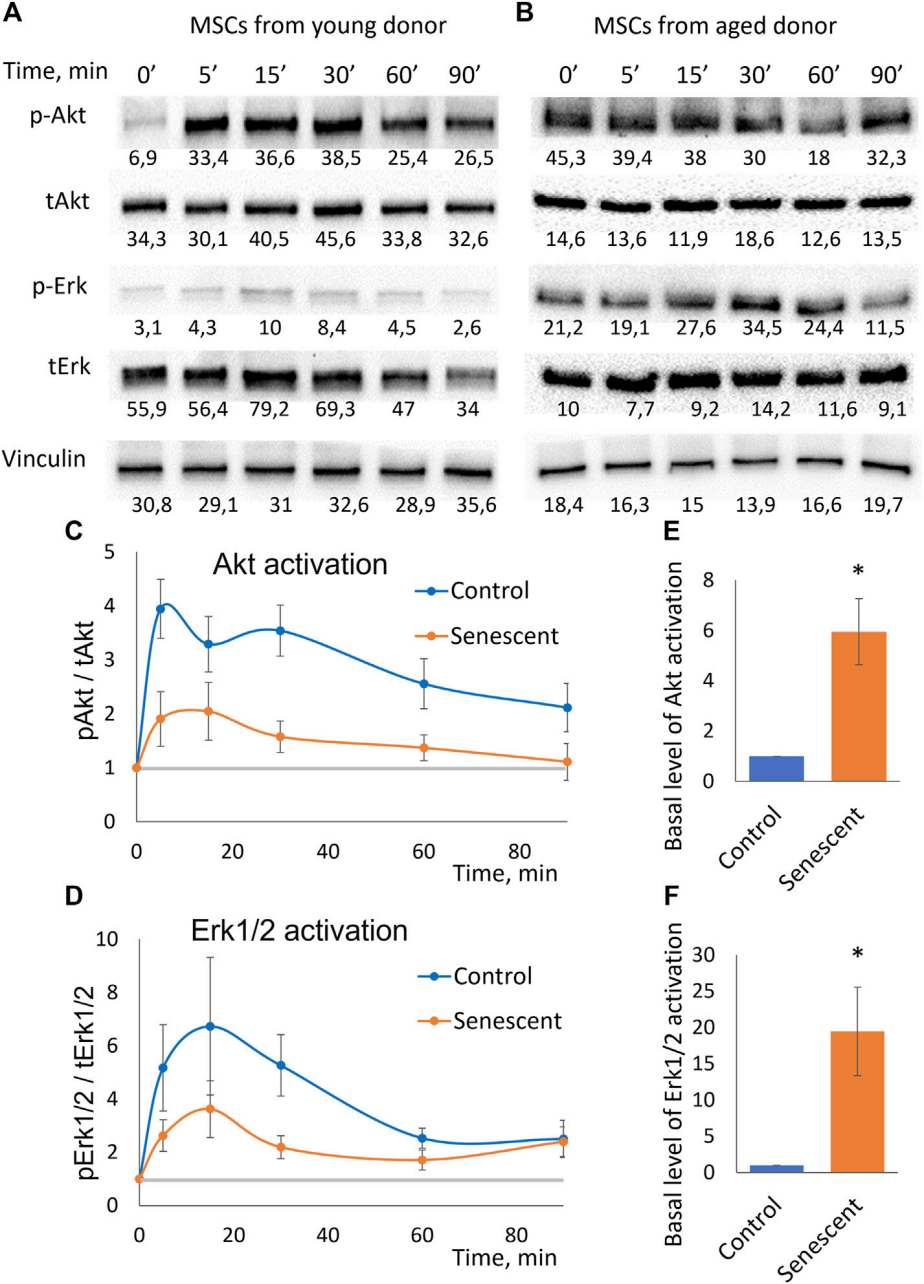
Insulin-dependent signaling in control and senescent MSCs. **(A,B)**. Representative results of Western-blot analysis of Akt (Thr308) and Erk1/2 phosphorylation under the action of insulin in different time points in control **(A)** and senescent **(B)** MSCs. The results of densitometric analysis marked under the protein bands; **(C)**. Time dynamics of Akt (Thr308) phosphorylation in control and senescent MSCs under insulin stimulation, *n* = 6 Y-axis represent ratio in Western-blot band volume between p-Akt (T308) and total Akt; **(D)**. Time dynamics of Erk1/2 (Thr202/Tyr204) phosphorylation in control and senescent cells under insulin stimulation, *n* = 6 Y-axis represent ratio in Western-blot band volume between p-Erk1/2 (Thr202/Tyr204) and total Erk; **(E,F)**. Basal level of Akt **(E)** and Erk1/2 **(F)** phosphorylation in control and senescent cells, *n* = 4–5. Mean ± SE, *p < 0.05.

To uncover the molecular mechanisms of decrease in adipogenic potential of senescent MSCs we studied their sensitivity to insulin. Using Western blotting analysis, we measured time dynamics of insulin-dependent phosphorylation of Akt (by Thr308) which indicates activation of PI3-kinase signaling cascade, and Erk1/2 mitogen-activated protein kinases (Figures 3A,B). We found that the insulin-dependent addition in phosphorylation of these kinases is sharply reduced in senescent cells (Figures 3C,D). Replicative cells showed a similar decrease in insulin-dependent signaling (Supplementary Figures S3–S5). The decrease in relative changes in the level of phosphorylation may be due to either a decrease in their insulin-dependent stimulation, or a high basal level of phosphorylation. The absolute level of phosphorylation of Akt and Erk1/2 did not change in senescent cells. In contrast, senescent MSCs demonstrated significantly increased levels of both Akt and Erk1/2 phosphorylation in non-stimulated cells (Figures 3B,E,F). As a result, the relative changes in the phosphorylation level were much weaker in senescent cells. Thus, senescent MSCs demonstrated resistance to insulin-dependent stimuli due to the high basal levels of Akt and Erk1/2 phosphorylation.

### Senescent MSCs secreted extracellular vesicles contained microRNA regulating cell response to metabolic and differentiation signals

**FIGURE 4:**
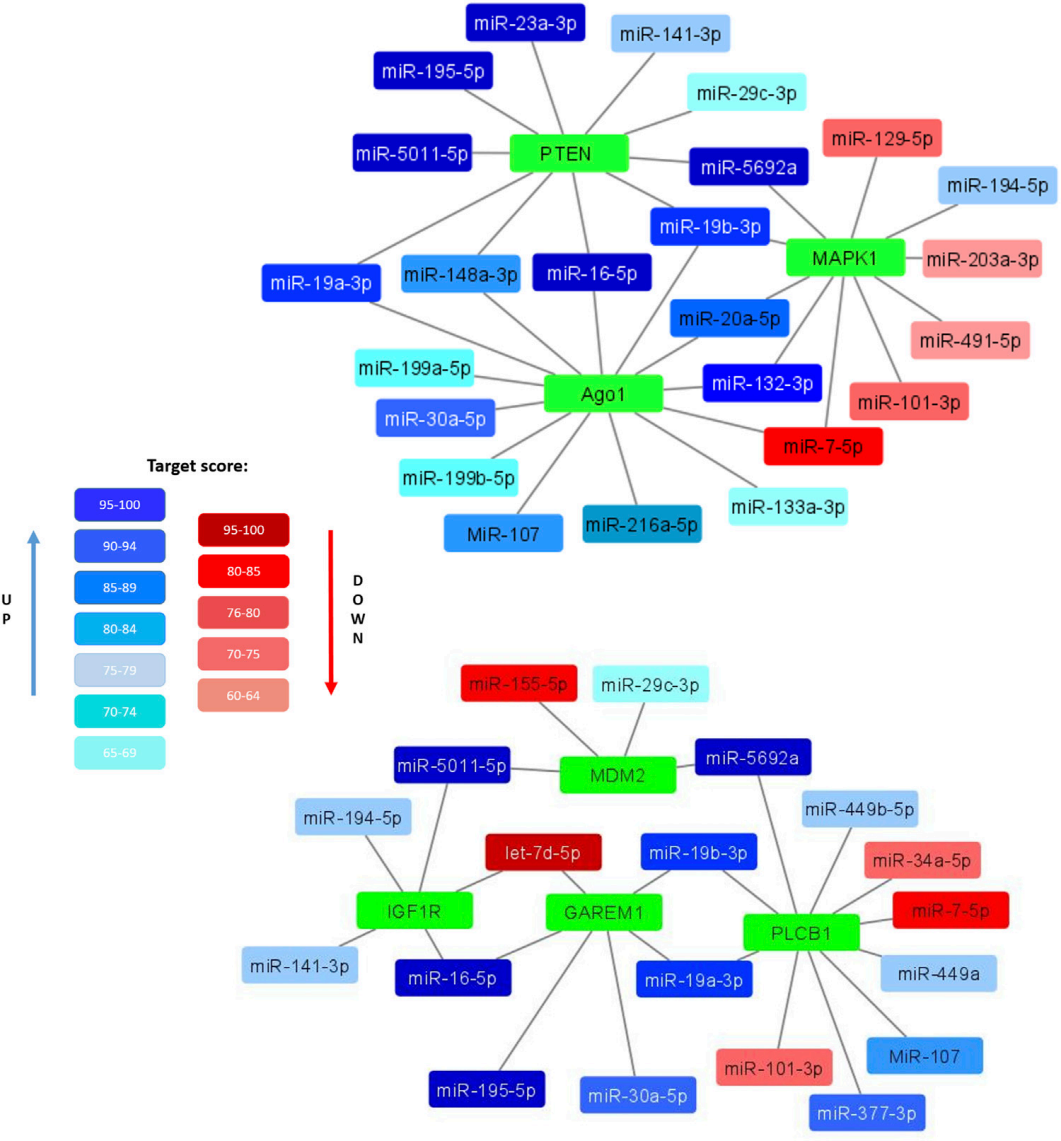
Selected miRNAs differentially expressed in EVs secreted by control and senescent MSCs along with their targets. Interaction maps illustrated differently expressed miRNAs as edges and their potential target mRNA as nodes. Coloured miRNAs are shown if significantly reduced (red) or enhanced (blue) in MSC-EVs from aged donors compared to young ones. The degree of color saturation shows the value of the target score level (relative units). A more saturated color indicates a higher value, and therefore a high reliability of the relationship between the miRNA and its target.

Clarifying the possible mechanisms of the observed decrease in the adipogenic potential and insulin resistance of senescent MSCs, we compared the composition of miRNAs in EVs secreted by MSCs from young and aged donors. We identified a number of miRNAs, whose expression level differed significantly. Bioinformatic analysis indicated that common targets for the miRNAs, which were significantly upregulated in EVs secreted by senescent MSCs, related to such processes as telomere maintenance, DNA repair and DNA damage response whereas downregulated miRNA targets were involved into the regulation of PI3-kinase and transforming growth factor beta (TGFb) signaling pathways. We also found a number of miRNAs unique to EVs secreted by senescent MSCs (miR-377–3p, miR-148–3p, miR-302b-3p, miR-141–3p). Among the most relevant predicted mRNA targets for the differently expressed miRNAs we detected mRNA encoding proteins involved in the metabolic responses of MSCs. In particular, we found the targets regulating insulin signaling and adipogenic differentiation (Figure 4):

• PTEN (phosphatase and tensin homolog) is phosphatidylinositol-3,4,5-trisphosphate 3-phosphatase, a negative regulator of PI3K/AKT insulin signaling pathway and inhibitor of glucose metabolism in adipose tissue;
• MAPK1 (mitogen-activated protein kinase 1, ERK2) is serine/threonine kinase, a component of the MAP kinase pathway which is an essential point for multiple signals in cells, including a key pathway for insulin activation of adipogenesis;
• MDM2 (mouse double minute 2 homolog/E3 ubiquitin-protein ligase) - an important regulator of insulin-dependent activation of PPARγ in adipocytes;
• GAREM1 (GRB2 associated regulator of MAPK1 subtype 1) is a positive regulator of MAPK pathway;
• IGF1R (insulin-like growth factor 1 receptor) is a receptor whose signaling pathways, like those of the insulin receptor, are involved in the regulation of cell proliferation and adipogenic differentiation;
• PLCB1 (1-Phosphatidylinositol-4,5-bisphosphate phospholipase beta-1) plays a role in controlling the cell cycle during adipogenic differentiation;
• Ago1 (argonaute RISC component 1)—a protein, playing an important role in RNA interference and RNA silencing, in particular, in the process of adipogenic differentiation.

All these targets are involved in the development and regulation of pro-adipogenic signals in cells. Major miRNAs involved in the regulation of these targets are miR-19a-3p, miR-19b-3p, miR-16–5p, miR-195–5p, miR-148a-3p, miR-20a-5p and miR-132–3p. Thus, EVs expressed by senescent cells can potentially be negative regulators of adipogenic differentiation of MSCs.

### Extracellular vesicles from senescent MSCs decreased the adipogenic potential of MSCs

**FIGURE 5:**
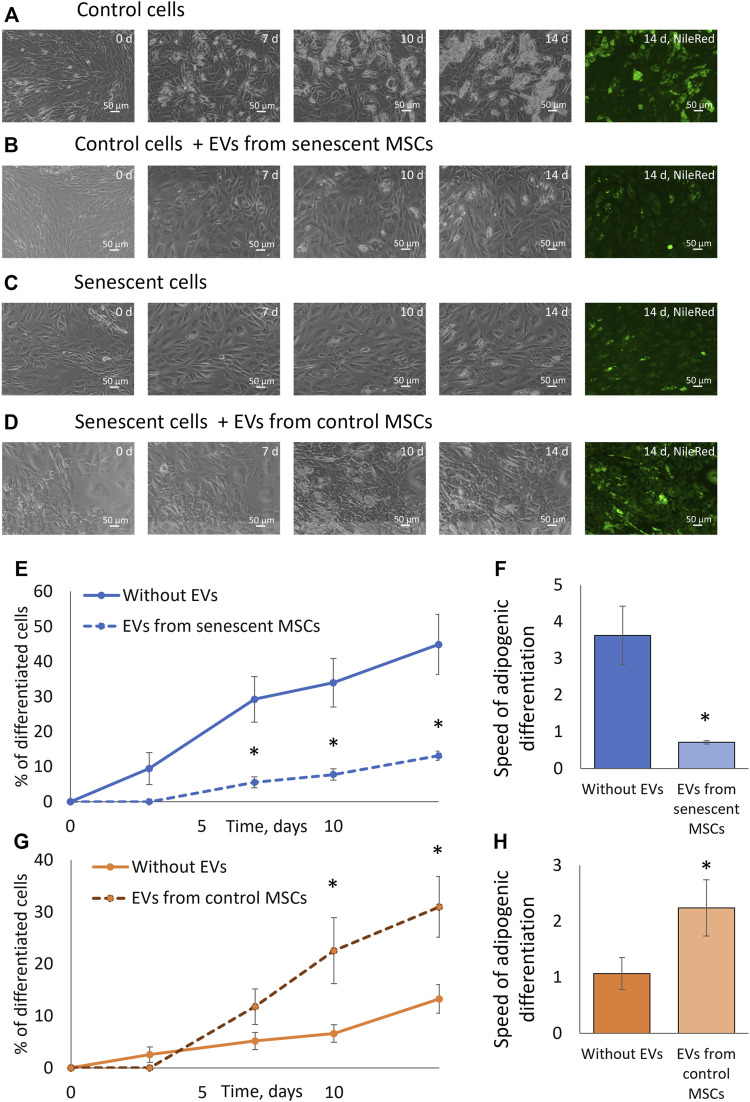
Extracellular vesicles affect the adipogenic potential of MSCs. **(A)**. Phase-contrast images of the dynamics of differentiation of control cells for 14 days with staining with neutral lipid dye NileRed on the 14th day; **(B)**. Phase-contrast images of the dynamics of differentiation of control cells with stimulation by EVs from senescent cells for 14 days with staining with neutral lipid dye NileRed on the 14th day; **(С)**. Phase-contrast images of the dynamics of differentiation of senescent cells for 14 days with staining with neutral lipid dye NileRed on the 14th day; **(D)**. Phase-contrast images of the dynamics of differentiation of senescent cells with stimulation by EVs from control cells for 14 days with staining with neutral lipid dye NileRed on the 14th day; **(E)**. Dynamics of increase in the number of cells accumulating fat drops during adipogenic differentiation of control MSCs in the presence of EVs from senescent cells and without, *n* = 7; **(F)**. The tangent of the slope of the increase in the number of differentiating control cells (differentiation rate) in the presence of EVs from senescent cells and without EVs, *n* = 7–11; **(G)**. Dynamics of increase in the number of cells accumulating fat drops during adipogenic differentiation of senescent MSCs in the presence of EVs from control cells and without EVs, *n* = 6; **(H)**. The tangent of the slope of the increase in the number of differentiating senescent cells (differentiation rate) in the presence of EVs from control cells and without, *n* = 5–9. Mean ± SE, *p < 0.05, **p < 0 01.

We examined the effect of extracellular vesicles and miRNAs contained in them on the adipogenic potential of MSCs. To do this, we treated control MSCs with EVs from senescent cells and, conversely, MSCs isolated from elderly donors were treated with EVs isolated from control cells. After treating the cells with vesicles for a day, we stimulated the cells to differentiate into adipocytes. At each change in the differentiation medium, we added a new portion of corresponding EVs. As can be seen from Figure 5, EVs secreted by senescent MSCs significantly reduce both the proportion of differentiating cells and the rate of adipogenic differentiation. EVs secreted by replicative MSCs showed the same effect (Supplementary Figures S6–S9). At the same time, EVs secreted by MSCs from young donors increase the adipogenic potential of MSCs isolated from old donors. EVs secreted by MSCs from aged donors or young donors do not significantly change the adipogenic potential of senescent or control MSCs, respectively (data not shown). Thus, EVs have a physiological effect on the adipogenic potential of MSCs, and the effect of EVs differs depending on their source.

## Discussion

During the lifespan, senescent cells are accumulated in different tissues, and stem cell senescence and replicative exhaustion are considered as hallmarks and promoters of aging and functional attrition in organisms (Rodier and Campisi 2011; van Deursen 2014; Childs et al., 2015). Particularly, these processes contribute to the age-associated dysfunction of adipose tissue promoting the development of adipose tissue insulin resistance, metabolic syndrome and type 2 diabetes as well as other concomitant serious diseases (reviewed in Tchkonia et al., 2010). Thus, Minamino et al. demonstrated that in adipose tissue p53 expression as a biomarker of senescence is crucially involved in the development of insulin resistance, which underlies age-related cardiovascular and metabolic disorders (Minamino et al., 2009). Experimental and clinical findings implicate cellular senescence as a causal factor in age- and obesity-related inflammation and metabolic derangements and provided the evidence that senolytic agents or other strategies targeting the senescent cells could be promising for treating metabolic dysfunction and its complications (Baker et al., 2011; Hickson et al., 2019; Palmer et al., 2019; Chaib et al., 2022). However, the cellular and molecular mechanisms underlying the relationships between adipose tissue dysfunction and senescence are still unclear. Especially, in the light of a constant renewal of adipose tissue throughout a person’s life (Arner et al., 2010). In our study we focused on the possible mechanisms of the impairment of adipose tissue renewal mediated by the senescence of adipose-derived MSCs, which represent the multipotent stem cell population differentiating in new adipocytes under the insulin stimuli.

Increasing evidence supports the hypothesis that cellular senescence recapitulates aspects of organism aging and contributes to aging phenotypes *in vivo*, in part by limiting self-renewal of tissues by stem and progenitor cells (Rando Thomas A. 2006; Sedivy JM et al., 2008; Rodier and Campisi 2011; Sikora et al., 2011; Mitterberger et al., 2014; van Deursen 2014). MSCs contain a subpopulation of multipotent stem cells, critically important for adipose tissue maintenance and regeneration. Additionally, MSCs are able to secrete a variety of regulatory factors including cytokines, growth factors, non-coding RNAs (mostly within EVs), extracellular matrix components, etc. MSC secretome could mediate the most of MSC regenerative effects being involved in the regulation of tissue-specific stem cells and their niche (Sagaradze et al., 2020). Senescence of MSCs causes crucial impairment of their regenerative capacity and functional persistence (Neri and Borzì 2020). Several studies demonstrate that MSC senescence is often associated with a decrease in the rate of adipose tissue renewal and subsequent adipose tissue hypertrophy. Interestingly, with the development of hypertrophic obesity and type 2 diabetes, the number of adipose MSCs does not decrease, but most of them exhibit a senescent phenotype (Gustafson et al., 2019).

Previously, Mitterberger et al. demonstrated that adipose-derived MSCs isolated from abdominal subcutaneous fat pads of adult donors acquired replicative senescence after long-term cultivation and it contributed to the dysfunctions in their replication, adipogenesis, triglyceride storage, and adipokine secretion (Mitterberger et al., 2014). In our study, we also observed that senescent MSCs, derived from aged donors or obtained by long-term cell culture up to replicative senescence, manifested a dramatic decrease in their adipogenic potential leading to the development of insulin resistance. We are first to show that the latter is due not to a decrease in the level of activation of insulin-dependent signaling cascades, but to a noticeable increase in basal level of Akt and Erk1/2 phosphorylation in cells. Thus, in our experiments we observed a significant increase in basal phosphorylation level of the insulin signaling effector kinase Akt in senescent MSCs. At the same time, level of Akt phosphorylation after insulin stimulation did not increase in senescent cells suggesting the condition of insulin resistance (Liu et al., 2009). Insulin-dependent Akt phosphorylation is crucial for adipogenic differentiation as it leads to the upregulation of adipogenic master-regulator PPARg resulting in expression of adipocyte-specific genes (Zhang et al., 2009). Dysregulation of this signaling pathway in senescent MSCs could be responsible for decreased adipogenic potential. Of note, senescence has a much smaller effect on activation of another insulin-activated signaling pathway, MAPK/ERK, in MSCs. This signaling pathway plays an important role in mediating the anabolic effects of insulin (Zhou et al., 2018). However, during MSC differentiation MAPK/ERK signaling is known to inhibit adipogenesis, while stimulating osteogenesis (Ge et al., 2016). Thus, another reason for decreased adipogenesis in senescent cells could be shift of balance between different signaling pathways.

The observation that senescent cells demonstrate not decreased ability to phosphorylate Akt, but increased basal level of phosphorylation could suggest that the main dysregulation of insulin signaling occurs on the level of specific repressors, such as PTEN (Jiang et al., 2020). Normally, PTEN acts as a repressor for hyperactivation of PI3K/Akt signaling pathway. However, during cell senescence it levels could change and affect both insulin sensitivity and adipogenic potential of cells (Kirstein et al., 2021).

To reveal the possible mechanisms of the observed effects we focused on intercellular communication mediated by their secretome components. Importantly, cellular senescence markedly affects MSC secretome leading to the development of SASP that comprises the release of proinflammatory cytokines, chemokines, growth factors and proteases as well as EVs with their cargo into the extracellular environment. EVs are important mediators of intercellular communication through the transfer of their contents such as proteins, mRNAs, microRNAs and DNA, and have been recognized as essential components of SASP that altered cells acquire during senescence. EVs secreted from senescent cells have distinctive characteristics, acting like SASP factors, they can affect the behavior of neighboring cells *via* autocrine/paracrine mechanisms leading to reprogramming of the microenvironment toward a pro-senescent state resulting in inflammation, stem cell dysfunction, and cancer progression (Xu and Tahara 2013; Kadota et al., 2018; Terlecki-Zaniewicz et al., 2018),. Similar results were obtained for EVs produced by senescent MSCs. Thus, it was shown that senescent MSC-EVs inhibited wound healing *via* a mechanism that involves downregulation of miR-146a (Xu et al., 2021). Kulkarni et al. (2018) showed that certain miRNAs within exosomes secreted by young MSCs can suppress cell aging of hematopoietic stem cells, whereas vesicles from senescent MSCs significantly aggravated this process (Kulkarni et al., 2018).

In our study we also demonstrated that EVs secreted by senescent MSCs attenuated the adipogenic differentiation of MSCs from young donors whereas EVs secreted by control MSCs restored the adipogenic potential of senescent MSCs. Moreover, we revealed some distinctive miRNA patterns within the EVs secreted by MSCs from aged donors. The bioinformatic analysis indicated their possible involvement in the regulation of senescence-associated processes (such as telomere attrition, proliferation, DNA damage response and DNA repair) as well as metabolic pathways and differentiation. Particularly, the predictive target of some miRNAs upregulated in EVs secreted by senescent MSCs is Ago1, a crucial factor for miRNA biogenesis pathway, which was recently demonstrated to be involved into the regulation of adipogenic differentiation (Martin et al., 2018). Few miRNAs were detected only in EVs secreted by MSCs from aged donors; among them miR-377 could regulate adipogenic differentiation of human MSC by targeting LIFR (Li et al., 2018). Wnt1 is also a target for miRNA-148a which could link it to the adipogenic differentiation (Shi et al., 2015). miR-20a significantly upregulated in EVs from senescent MSCs was shown to regulate adipocyte differentiation by targeting lysine-specific demethylase 6b and TGFb signaling (Zhou et al., 2015).

It should be noted that EVs produced by senescent cells contain several miRNAs that specifically target the regulators of insulin signaling pathway such as PTEN, MAPK1, MDM2, and others (Olive et al., 2009; Chakraborty et al., 2014). These findings may explain why in senescent cells an increase of basal level of Akt phosphorylation can be observed, but the specific targets for the revealed miRNAs should be further validated to confirm their contribution into senescence-associated decline of MSC adipogenic potential and insulin resistance.

## Conclusion

Taken together, we demonstrated that senescent MSCs or at least a subpopulation of these cells could attenuate the adipogenic potential of postnatal stem cells within adipose tissue due to the production of extracellular vesicles with altered pattern of microRNAs regulating the crucial components of insulin signaling pathways. These effects are strongly associated with insulin resistance development in senescent MSCs and could be partially reversed by modifying the secretory microenvironment. The specific molecular mechanisms mediating the autocrine and/or paracrine interactions of senescent MSCs and their contribution into adipose tissue renewal during aging should be revealed in further experiments.

## Data Availability

The original contributions presented in the study are included in the article/Supplementary Material, further inquiries can be directed to the corresponding authors.
