# Corrigendum: Declined adipogenic potential of senescent MSCs due to shift in insulin signaling and altered exosome cargo

**DOI:** 10.3389/fcell.2023.1146895

**Published:** 2023-01-31

**Authors:** Elizaveta Voynova, Konstantin Kulebyakin, Olga Grigorieva, Ekaterina Novoseletskaya, Natalia Basalova, Natalia Alexandrushkina, Mikhail Arbatskiy, Maxim Vigovskiy, Anna Sorokina, Anna Zinoveva, Elizaveta Bakhchinyan, Natalia Kalinina, Zhanna Akopyan, Vsevolod Tkachuk, Pyotr Tyurin-Kuzmin, Anastasia Efimenko

**Affiliations:** ^1^ Faculty of Medicine, Lomonosov Moscow State University, Moscow, Russia; ^2^ Institute for Regenerative Medicine, Medical Research and Education Center, Lomonosov Moscow State University, Moscow, Russia

**Keywords:** senescence, extracellular vesicles (EVs), insulin signaling, adipogenic potential, MSCs (mesenchymal stromal cells)

In the published article, there was an error in the **Funding** statement. The funding statement for the Russian Foundation for Basic Research was displayed as “project number 19-315-04172”. The correct **Funding** statement appears below.

Funding

“This reported study was funded by the Russian Foundation for Basic Research, project number 19-29-04172 (adipogenic differentiation, extracellular vesicles and miRNA analysis) and Russian Science Foundation, project number 21-15-00311 (Western-Blot analysis of intracellular signaling), and supported by the Development Program of the Interdisciplinary Scientific and Educational School of Lomonosov MSU “Molecular technologies of the living systems and synthetic biology”. Experiments were conducted using equipment purchased as part of Lomonosov MSU Development Program”.

The authors apologize for this error and state that this does not change the scientific conclusions of the article in any way. The original article has been updated.

