# Supplementary material for: Declined adipogenic potential of senescent MSCs due to shift in insulin signaling and altered exosome cargo
Source: Front Cell Dev Biol. 2022 Nov 17;10:1050489. doi: 10.3389/fcell.2022.1050489 (PMC9714334; doi:10.3389/fcell.2022.1050489)
Supplement: Supplementary file 1 [file Presentation1.pptx]

## Slide 1
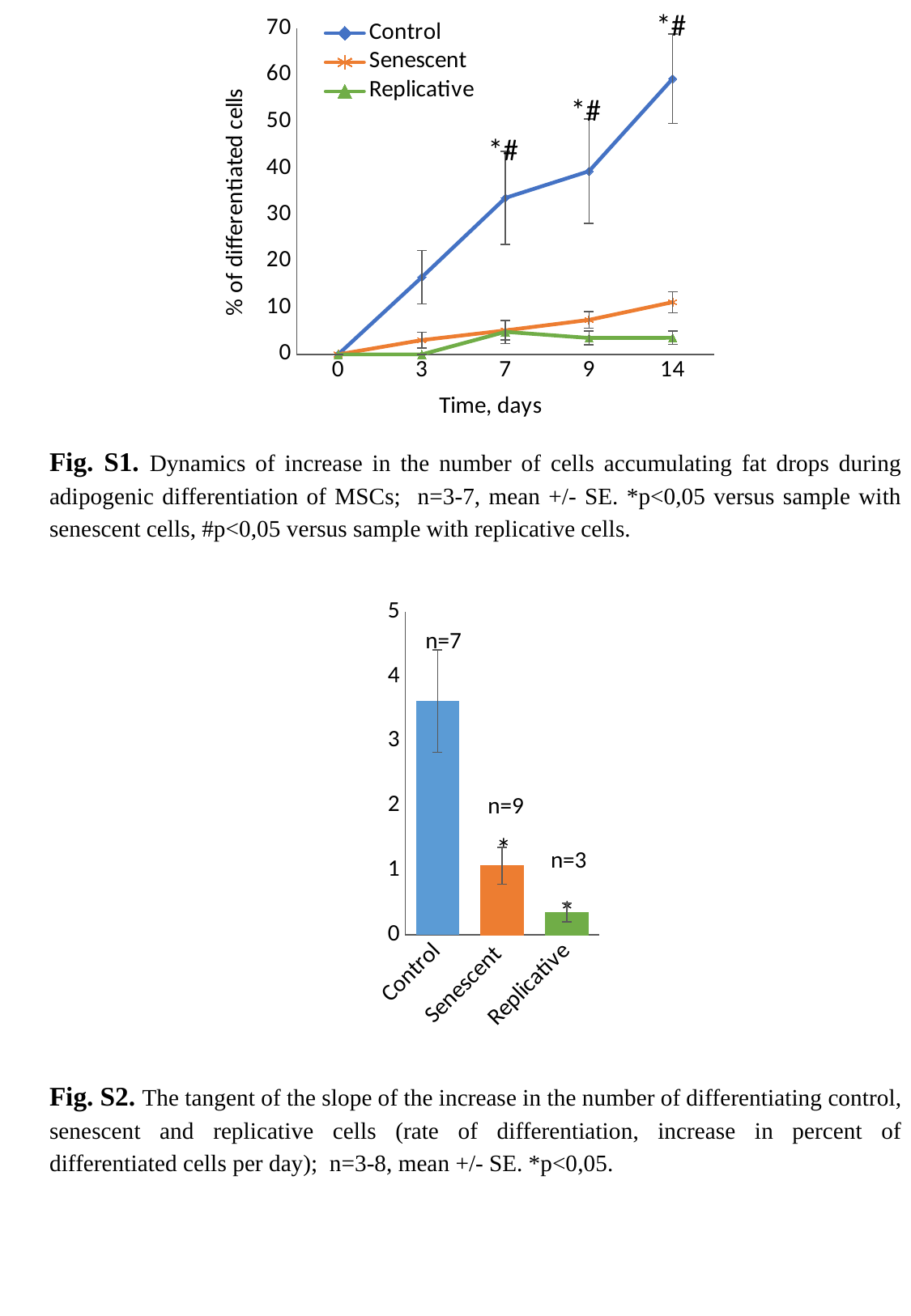

*#
### Chart
| Category | | | |
|---|---|---|---|
| 0 | 0.0 | 0.0 | 0.0 |
| 3 | 16.5875 | 3.0599999999999996 | 0.0 |
| 7 | 33.611363636363635 | 5.1508988764044945 | 4.839865840566412 |
| 9 | 39.34962121212121 | 7.415536420461794 | 3.5251151762419366 |
| 14 | 59.22183794466403 | 11.223718805480763 | 3.559624717688847 |*#
*#
Fig. S1. Dynamics of increase in the number of cells accumulating fat drops during adipogenic differentiation of MSCs; n=3-7, mean +/- SE. *p<0,05 versus sample with senescent cells, #p<0,05 versus sample with replicative cells.
### Chart
| Category | |
|---|---|
| Control | 3.6214285714285714 |
| Senescent | 1.0675000000000001 |
| Replicative | 0.3446333333333333 |n=7
n=9
*
n=3
*
Fig. S2. The tangent of the slope of the increase in the number of differentiating control, senescent and replicative cells (rate of differentiation, increase in percent of differentiated cells per day); n=3-8, mean +/- SE. *p<0,05.

## Slide 2
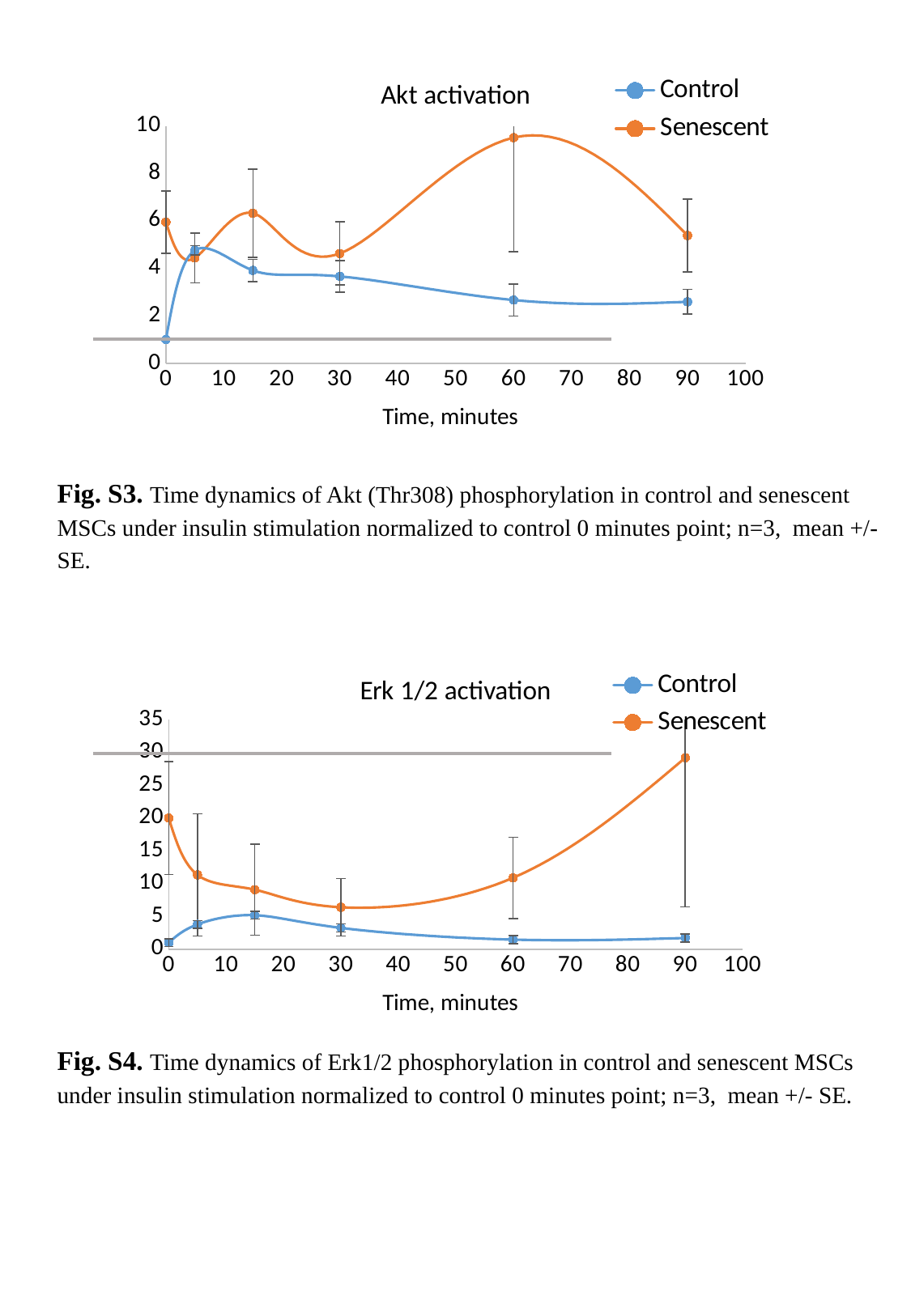

### Chart: Akt activation
| Category | | |
|---|---|---|Fig. S3. Time dynamics of Akt (Thr308) phosphorylation in control and senescent MSCs under insulin stimulation normalized to control 0 minutes point; n=3, mean +/- SE.
### Chart: Erk 1/2 activation
| Category | | |
|---|---|---|Fig. S4. Time dynamics of Erk1/2 phosphorylation in control and senescent MSCs under insulin stimulation normalized to control 0 minutes point; n=3, mean +/- SE.

## Slide 3
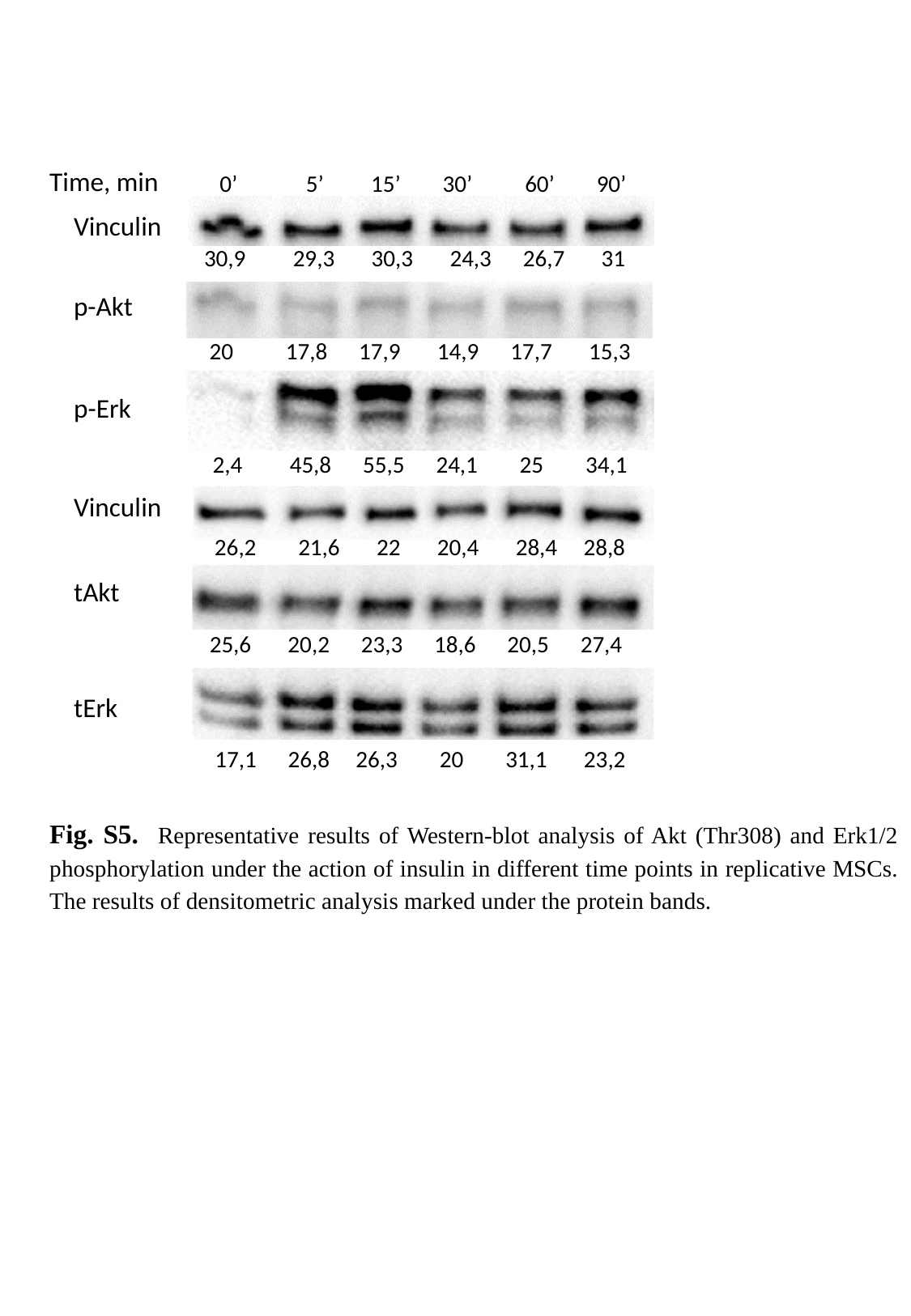

Time, min
 0’ 5’ 15’ 30’ 60’ 90’
Vinculin
 30,9 29,3 30,3 24,3 26,7 31
p-Akt
 20 17,8 17,9 14,9 17,7 15,3
p-Erk
 2,4 45,8 55,5 24,1 25 34,1
Vinculin
 26,2 21,6 22 20,4 28,4 28,8
tAkt
 25,6 20,2 23,3 18,6 20,5 27,4
tErk
 17,1 26,8 26,3 20 31,1 23,2
Fig. S5. Representative results of Western-blot analysis of Akt (Thr308) and Erk1/2 phosphorylation under the action of insulin in different time points in replicative MSCs. The results of densitometric analysis marked under the protein bands.

## Slide 4
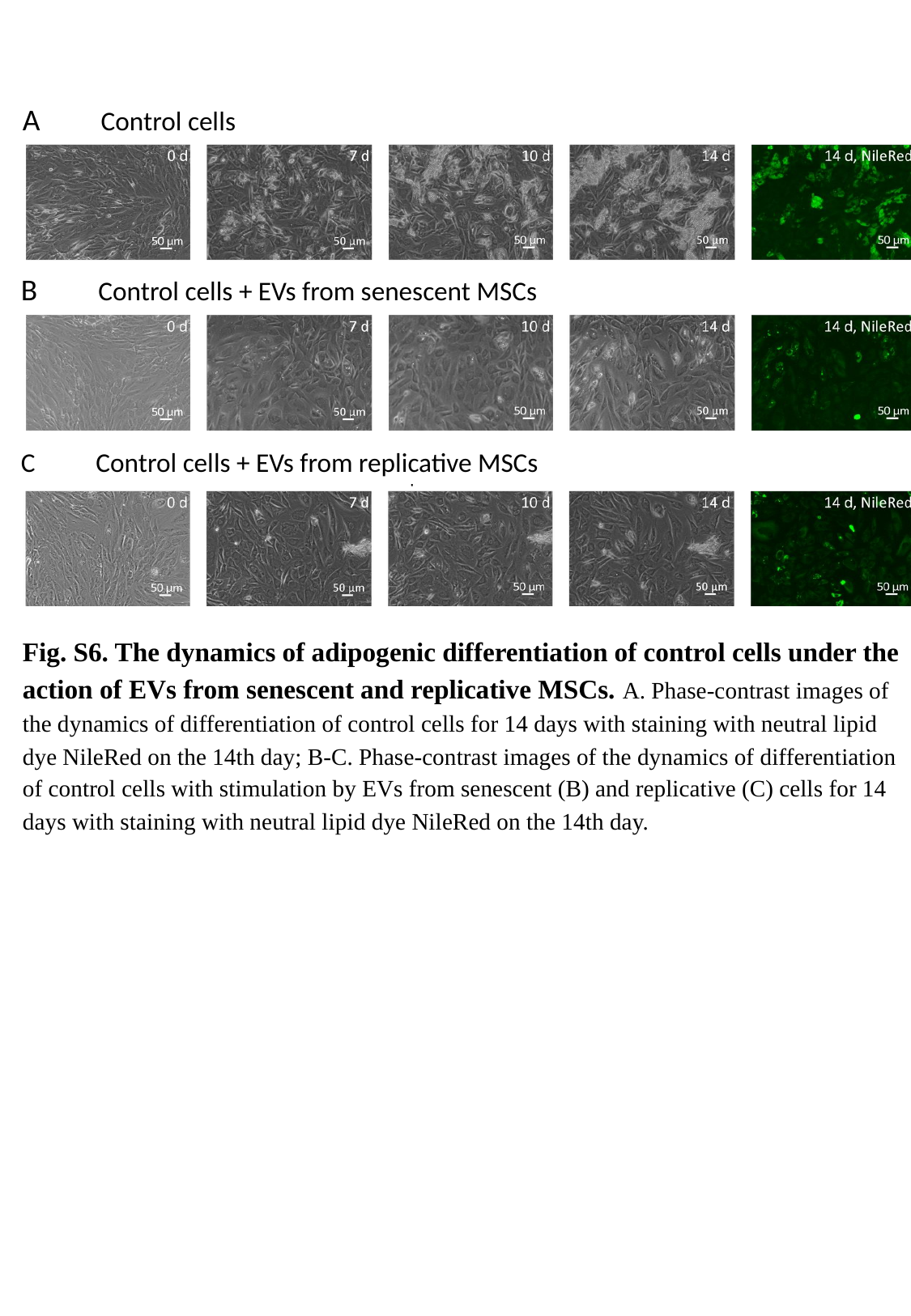

#
A Control cells
B Control cells + EVs from senescent MSCs
C Control cells + EVs from replicative MSCs
Fig. S6. The dynamics of adipogenic differentiation of control cells under the action of EVs from senescent and replicative MSCs. A. Phase-contrast images of the dynamics of differentiation of control cells for 14 days with staining with neutral lipid dye NileRed on the 14th day; B-C. Phase-contrast images of the dynamics of differentiation of control cells with stimulation by EVs from senescent (B) and replicative (C) cells for 14 days with staining with neutral lipid dye NileRed on the 14th day.

## Slide 5
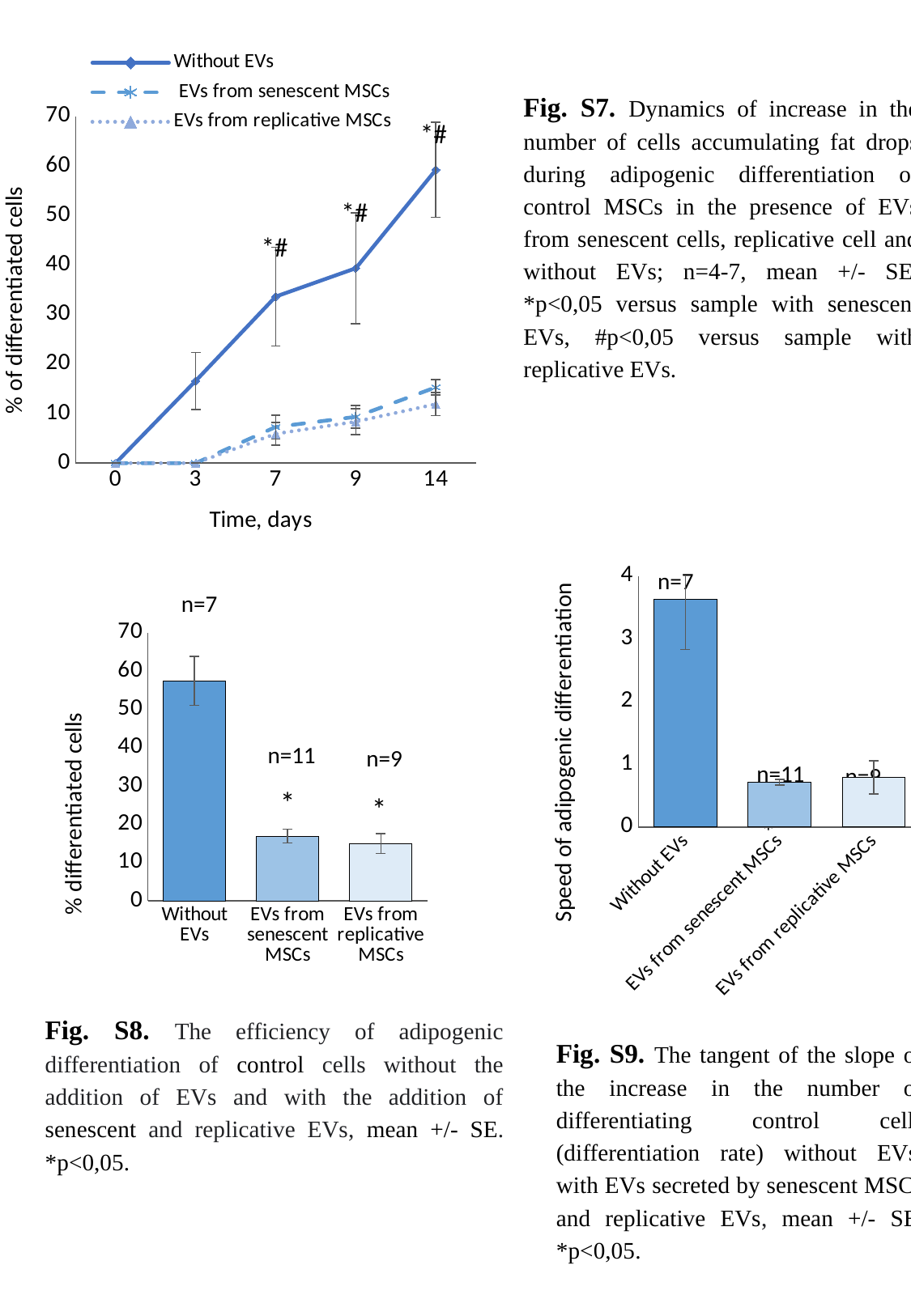

### Chart
| Category | Without EVs | EVs from senescent MSCs | |
|---|---|---|---|
| 0 | 0.0 | 0.0 | 0.0 |
| 3 | 16.5875 | 0.0 | 0.0 |
| 7 | 33.611363636363635 | 7.297293133784906 | 5.91935278241466 |
| 9 | 39.34962121212121 | 9.345550728646964 | 8.372591726792287 |
| 14 | 59.22183794466403 | 15.309046947298288 | 11.908253960652345 |*#
*#
*#
Fig. S7. Dynamics of increase in the number of cells accumulating fat drops during adipogenic differentiation of control MSCs in the presence of EVs from senescent cells, replicative cell and without EVs; n=4-7, mean +/- SE. *p<0,05 versus sample with senescent EVs, #p<0,05 versus sample with replicative EVs.
### Chart
| Category | |
|---|---|
| Without EVs | 57.42 |
| EVs from senescent MSCs | 16.906160628840862 |
| EVs from replicative MSCs | 14.968036491556406 |n=7
n=11
n=9
*
*
### Chart
| Category | |
|---|---|
| Without EVs | 3.6214285714285714 |
| EVs from senescent MSCs | 0.7112499999999999 |
| EVs from replicative MSCs | 0.7933333333333334 |n=7
n=11
n=9
*
*
Fig. S8. The efficiency of adipogenic differentiation of control cells without the addition of EVs and with the addition of senescent and replicative EVs, mean +/- SE. *p<0,05.
Fig. S9. The tangent of the slope of the increase in the number of differentiating control cells (differentiation rate) without EVs, with EVs secreted by senescent MSCs and replicative EVs, mean +/- SE. *p<0,05.

## Slide 6
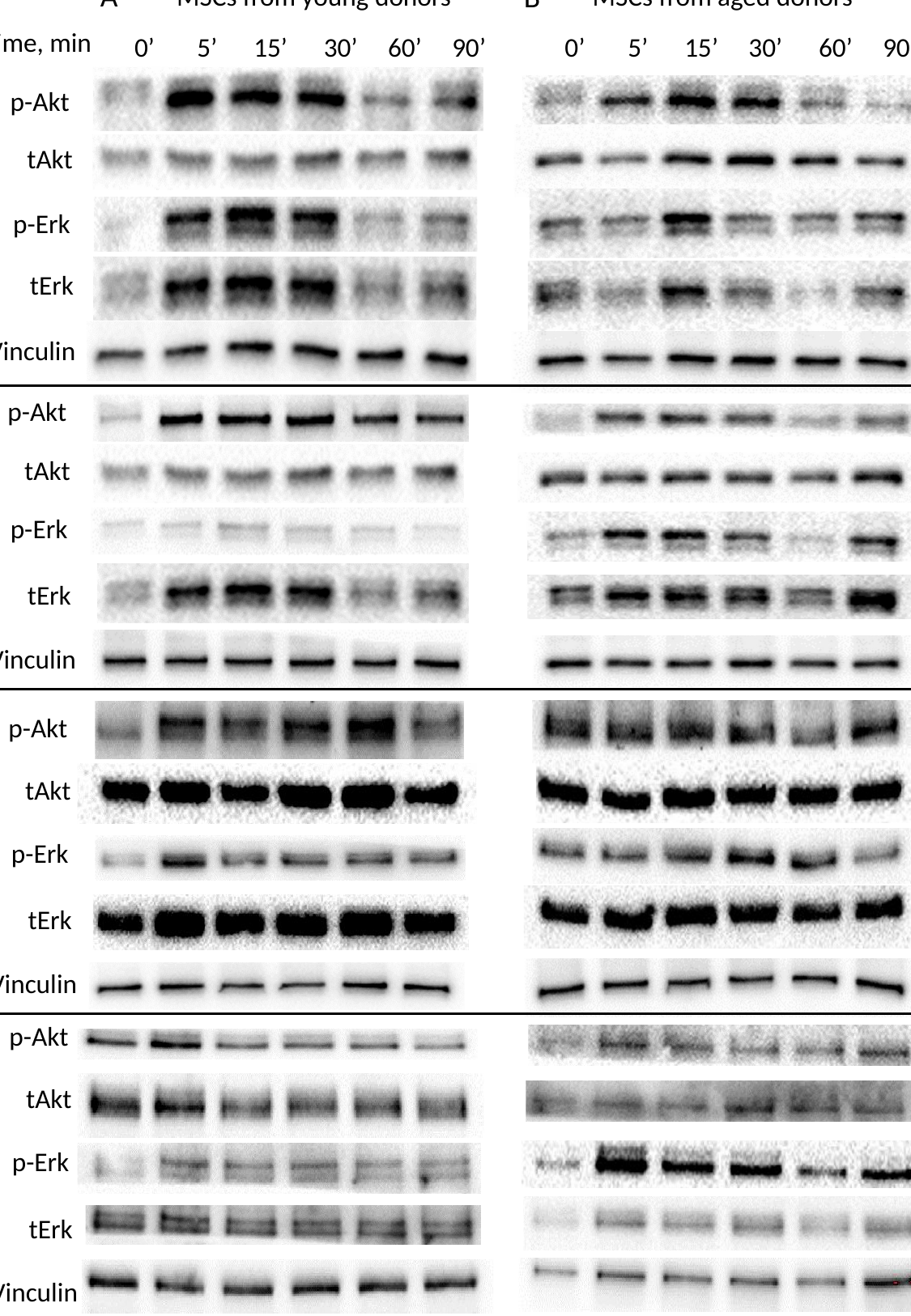

A
B
MSCs from young donors
MSCs from aged donors
Time, min
 0’ 5’ 15’ 30’ 60’ 90’ 0’ 5’ 15’ 30’ 60’ 90’
p-Akt
tAkt
p-Erk
tErk
Vinculin
p-Akt
tAkt
p-Erk
tErk
Vinculin
p-Akt
tAkt
p-Erk
tErk
Vinculin
p-Akt
tAkt
p-Erk
tErk
Vinculin

## Slide 7
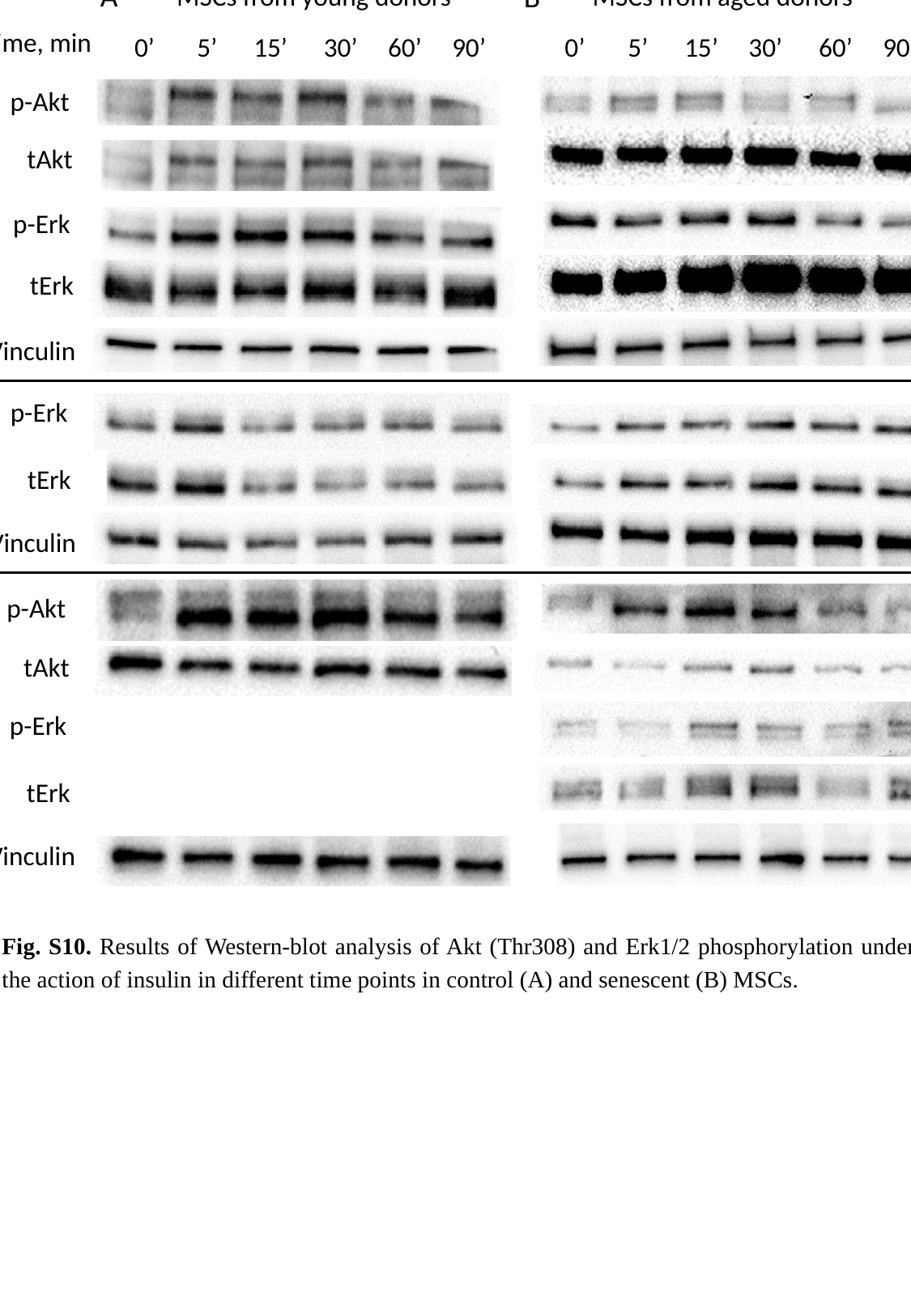

A
B
MSCs from young donors
MSCs from aged donors
Time, min
 0’ 5’ 15’ 30’ 60’ 90’ 0’ 5’ 15’ 30’ 60’ 90’
p-Akt
tAkt
p-Erk
tErk
Vinculin
p-Erk
tErk
Vinculin
p-Akt
tAkt
p-Erk
tErk
Vinculin
Fig. S10. Results of Western-blot analysis of Akt (Thr308) and Erk1/2 phosphorylation under the action of insulin in different time points in control (A) and senescent (B) MSCs.
